# Long-term thyroid hormone replacement therapy

**DOI:** 10.1186/1756-6614-6-S2-A53

**Published:** 2013-04-05

**Authors:** Jerzy Sowiński

**Affiliations:** 1 Department of Endocrinology, Metabolism and Internal Diseases, Poznan University of Medical Sciences, Poznan, Poland

## 

In recent years the number of patients with hypothyroidism has significantly increased. It is the result of increased prevalence of autoimmune thyroid disease, increased frequency of total thyroid resections and also common radioiodine thyroid ablation in patients with hyperthyroidism or differentiated thyroid cancers.

The treatment of hypothyroidism is considered by many physicians as effortless, simply based on L-thyroxine substitution. Universal access to therapy and to laboratory tests supports their approach. Is it true?

Nowadays, both the development of diagnostic methods and current knowledge concerning pathomechanism of thyroid disorders enable to establish a diagnosis much earlier, in subclinical phase, before typical symptoms appear. The question is, whether a patient should be treated at this early stage. If a doctor decides to start a hormonal therapy, next questions concerning a dose of medication and treatment monitoring must be answered.

The dose of L-thyroxine should be individually adjusted, based on both clinical and laboratory findings. Moreover, the age of patient, weight, physical activity, profession and general health state should be borne in mind. It is widely established, that the monitoring of TSH level is sufficient. However, in many cases even if TSH level is within the reference range, the patient's complaints, symptoms and quality of life are not satisfying. Therefore, assessment of other parameters of hormonal balance might be helpful.

Observations of patients treated for hypothyroidism bring new clinical dilemmas, unresolved despite of long-term experience. There are still questions, whether the laboratory test should be performed before or after taking medication? Should L-thyroxine be taken on the empty stomach every morning, or there are other options? How should results of hormonal tests be interpreted? What factors modify the therapeutic approach (age, pregnancy, coexisting disorders, elective surgery and other)?

To conclude, the treatment of hypothyroidism requires multifaceted approach. Therefore, medical care for patients cannot be limited only to monitoring of TSH level, what unfortunately is common in medical practice.

